# Runx3 Expression Inhibits Proliferation and Distinctly Alters mRNA Expression of Bax in AGS and A549 Cancer Cells

**Published:** 2011

**Authors:** Maryam Torshabi, Mohammad Ali Faramarzi, Mojtaba Tabatabaei Yazdi, Seyyed Naser Ostad, Mohammad Hosein Gharemani

**Affiliations:** a *Department of Pharmaceutical Biotechnology, Biotechnology Research Centre, Faculty of Pharmacy, Tehran University of Medical Sciences, Tehran, Iran. *; b *Department of Pharmacology and Toxicology, Faculty of Pharmacy, Tehran University of Medical Sciences, Tehran, Iran.*

**Keywords:** Runt, Cancer cells, Proliferation, Cell death, MRNA

## Abstract

Runx3, a member of Runt-related transcription factor (Runx) proteins with tumor suppressor effect, is a tissue–restricted and cancer related transcription factor that regulate cell proliferation and growth, as well as differentiation. In the present study, exogenous Run3 was transiently expressed in AGS (human gastric adenocarcinoma), with undetectable Runx3 protein and in A549 (human lung carcinoma) with low levels of endogenous Runx3 protein.

The GFP tagged Runx3 was transfected into AGS and A549 cells using fugene6 and PolyFect and Runx3 expression was confirmed by fluorescent microscopy and RT-PCR. The effect of Runx3 transfection on cell proliferation was determined by MTT assay and the results were confirmed by the trypan blue dye exclusion method. The effect of Runx3 expression on mRNA expression of BCL2-associated X protein (Bax) was evaluated using RT-PCR.

In AGS and A549 cells, Runx3 expression inhibited cell proliferation (p < 0.01). The growth inhibition was less in A549 cells. We show that Runx3 expression increases Bax mRNA expression in AGS cells when compared with control (p < 0.05), but no significant differences in mRNA expression was observed in both examined cells.

Runx3 expression has antiproliferative effect in AGS cell perhaps via increase in expression of Bax. The effect of Runx3 on A549 cells’ viability which has endogenous level of Runx3 is not related to Bax. These findings implicate a complex regulation by Runx3 in inhibition of cell proliferation utilizing Bax.

## Introduction

Runx (Runt-related) family of genes is DNA-binding and cancer related transcription factors that regulate the cell growth, proliferation and differentiation in humans (1, 2). The three members of this family are important targets of transforming growth factor *β* (TGF-*β*) super family signaling and play critical functions in mammalian development (3). Runx proteins have shown to interact with downstream SMAD (small mothers against decapentaplegic) proteins in mediating the growth-suppressive effects of TGF-*β* (4). Although all three members of Runx family share highly conserved DNA-binding domains (128 amino acid regions designated as runt domain), they regulate distinct functions and play important roles in both normal developmental processes and carcinogenesis (5-7). Runx1 is involved in hematopoiesis, angiogenesis and human acute leukemia (8) and Runx2 is essential for bone and tooth development and involved in cleidocranial dysplasia (9).

Runx3, the smallest member of Runx family, is a putative tumor suppressor gene located at chromosome 1p36 and is critical for gastric epithelial differentiation, neurogenesis of dorsal root ganglia and T cell differentiation (10, 11). In addition, Runx3 is deeply involved in various cancer processes such as cell growth, apoptosis, angiogenesis, and metastasis (12). Previous studies strongly suggested that Runx3 is a tumor suppressor in various carcinomas, including gastric and breast carcinoma and its loss is related to frequently inactivation by dual mechanism of protein cytoplasmic mislocalization and promoter hypermethylation (10, 12-15). Interestingly, the oncogenic potential of Runx3 has also been observed in some cases, such as head and neck and ovarian cancer, due to the demethylation and/or cytoplasmic mislocalization (12, 16, 17). 

Runx3 plays an important role in inhibiting cellular growth by participating in the TGF-*β* signaling pathway. It has been shown that Runx3 is responsible for transcriptional up-regulation of Bim in TGF-*β*-induced apoptosis (14, 18). Therefore, expression of exogenous Runx3 and study of its effect on cell proliferation and cell death can provide important information to develop anticancer agents and gene therapy strategies as well as better diagnosis. 

In this study, we have evaluated the over-expression effect of Runx3 on proliferation and viability of cells with undetectable Runx3 expression (AGS) and low Runx3 expression (A549). Furthermore, we tested the effect of Runx3 expression on mRNA expression of Bax in these cells.

## Experimental

*Cell culture and transfection*

The human lung cancer cell line A549 and human gastric adenocarcinoma cell line AGS (Pasteur Institute Cell Bank, Iran) were grown in RPMI-1640 medium (Euroclone, EU) supplemented with 10% fetal bovine serum (Gibco, UK) and 1% antibiotic penicillin-streptomycin (Biosera, UK) in 5% CO_2_ at 37^°^C incubator.

Human Runx3 complementary DNA (cDNA) (NM_004350) was subcloned into pReceiver-EGFP vector (Genecopoeia, USA) and transiently transfected into A549 and AGS cell lines. Cells were plated 24 h before transfection to reach 70-90% confluency. After 24 h, A549 cells and AGS cells were transfected with Fugene^®^ 6 reagent (Roche, Germany) and PolyFect^®^ transfection reagent (Qiagen, Germany), respectively, according to the instruction manual. The untransfected cells and the cells transfected with empty vector were used as negative controls. The transfected cells were analyzed for Runx3 protein expression using EGFP (enhanced green fluorescent protein) tag by fluorescent microscopy at various time points. The Runx3 gene expression was further analyzed using western blot analysis.

*Western blot analysis*

To detect Runx3 protein expression, the cells were transfected and cell lysates were prepared from attached and float cells 24, 48 and 72 h after the transfection. Standard western blotting was done with a polyclonal antibody against human Runx3 (Santa Cruz, USA). 

*Cell proliferation assays*

The cell proliferation and cell viability were evaluated 24 and 48 h after transiently transfecting using Methyl tetrazolium cytotoxicity assay (MTT, Sigma, UK) and Trypan blue dye exclusion method. For measuring cell viability, cells were plated in 96-well plates at density of 2.5x10^4^ cells/well in triplicate. Cells were incubated, 24 and 48 h after the transfection, with MTT solution (3-(4, 5-dimethyl thiazol-2-yl)-2, 5-diphenyl tetrazolium bromide, 0.5 mg/mL) for 4 h at 37°C. After the incubation period, the formazan crystals were dissolved in DMSO. The optical density (OD) was measured on microplate reader (BioTek, USA) at 570 nm, with 690 nm as a reference wavelength. The change in viability was calculated as percent viability compared to control group and the results of the three independent experiments were presented as mean ± SE (n = 3). 

To evaluate the number of viable cells, the cells were seeded at a density of 1×10^5 ^cells/well into 24-well culture plates in triplicate. The number of live cells in each well was counted at 24 and 48 h after transfection using 0.4% trypan blue dye (Sigma, USA). The change in live cells was calculated as control percentage and results of the three independent experiments were presented as mean ± SE (n = 3). 

*Semiquantitative analysis of Bax MRNA levels by RT-PCR*

Total RNA was extracted from A549 and AGS cells 24 h after transfection using TriPure Isolation Reagent^®^ (Roche, Germany) and cDNA was synthesized from 2 µg of total RNA by using RevertAid^®^ H Minus Kit (Fermentas, EU ) according to the manufacturer^’^s manual. 

BCL2-associated X protein (Bax) mRNA (Messenger RNA) expression was measured by the semiquantitative reverse transcriptase-polymerase chain reaction (RT-PCR) at different concentrations of cDNA (0.02, 0.08 and 0.16 µg/µL) using 5′- CAGCTCTGAGCAGATCATGAAGAC -3′ (sense) and 5′- GCCCATCTTCTTCCAGATGGTGAGC -3′ (antisense) primers, which yielded a 535 bp PCR product (19). The PCR was performed using the following program: 95^°^C for 2 min, 35 cycles of denaturation at 95^°^C for 30 sec, annealing at 64^°^C for 30 sec, and extension at 7°C for 40 sec and a final extension at 72°C for 5 min. The *β*-actin was used as house-keeping gene internal control (20). The PCR products were analyzed on 1% agarose gels and UV illumination. The bands were digitized using Scion-Image software (Scion Corporation, USA). The expression ratio was calculated based on the band intensity of targeted gene to *β*-actin. 

*Statistical analysis*

Data presented as the means ± SE to at least three independent experiments. Statistical analysis was performed using one-way ANOVA followed by Tukey-Kramer’s multiple comparison and p-values less than 0.05 were considered as significant.

## Results

*Effect of Runx* *3* *expressionin AGS and A* *549* *cell proliferation*

**Figure 1:**
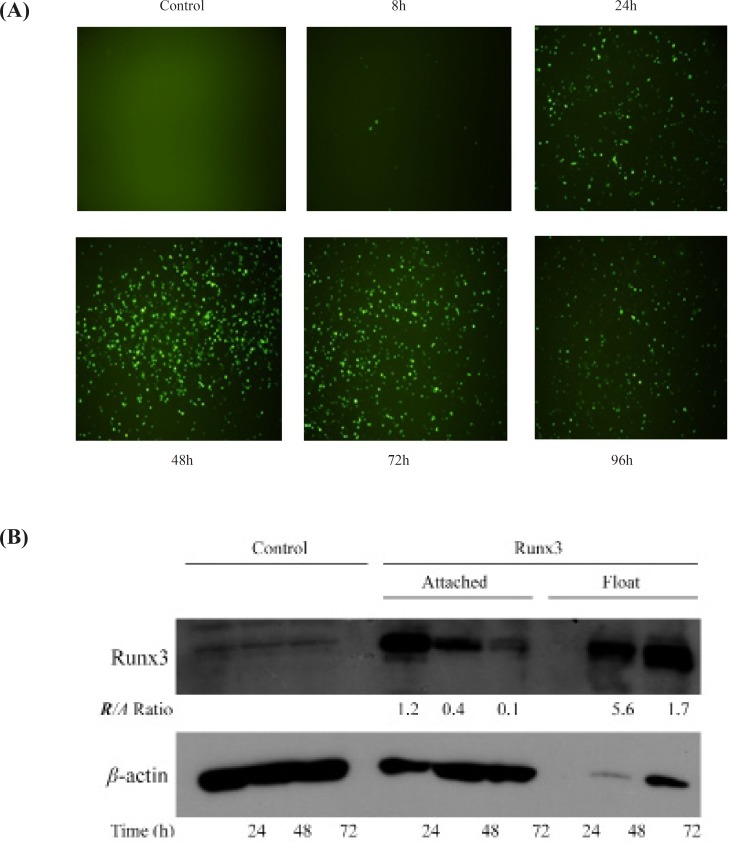
The expression of Runx3 in AGS cells AGS cells were transfected with Runx3/EGFP and compared with untransfected cells (Control). (A) The green fluorescent of EGFP expression was evaluated 8-96 h after transfection. (B) Runx3 protein expression was determined 24, 48 and 72 h after transfection by western blotting analysis in attached and floating cells. *β*-actin was used as internal control and expression ratios of Runx3 to *β*-actin (R/A) are shown

Transient transfection of Runx3 indicated the protein expression within 24 h intransfected cells confirmed by microscopic analysis of EGFP tag (Figure 1A). Furthermore, Western blotting analysis indicated the high protein expression of Runx3 after transfection (Figure 1B). Interestingly, most of the AGS cells expressing GFP -Runx3 were dead and floating. The western blot analysis indicated the over-expression of GFP- Runx3 in AGS cells. The expression was higher in dead floating cells compared to the attached cells (Figure 1B) . In A549 cells, unlike AGS cells, fewer transfected cells expressing GFP were detected. This difference can be due to the cell line variation, protein expression and/or the timing of cell death in these cells. As indicated , the expression of Runx3 washighin 24-72 hand decreased in 96 h due to the death of expressing cells (Figure 1A). 

**Figure 2:**
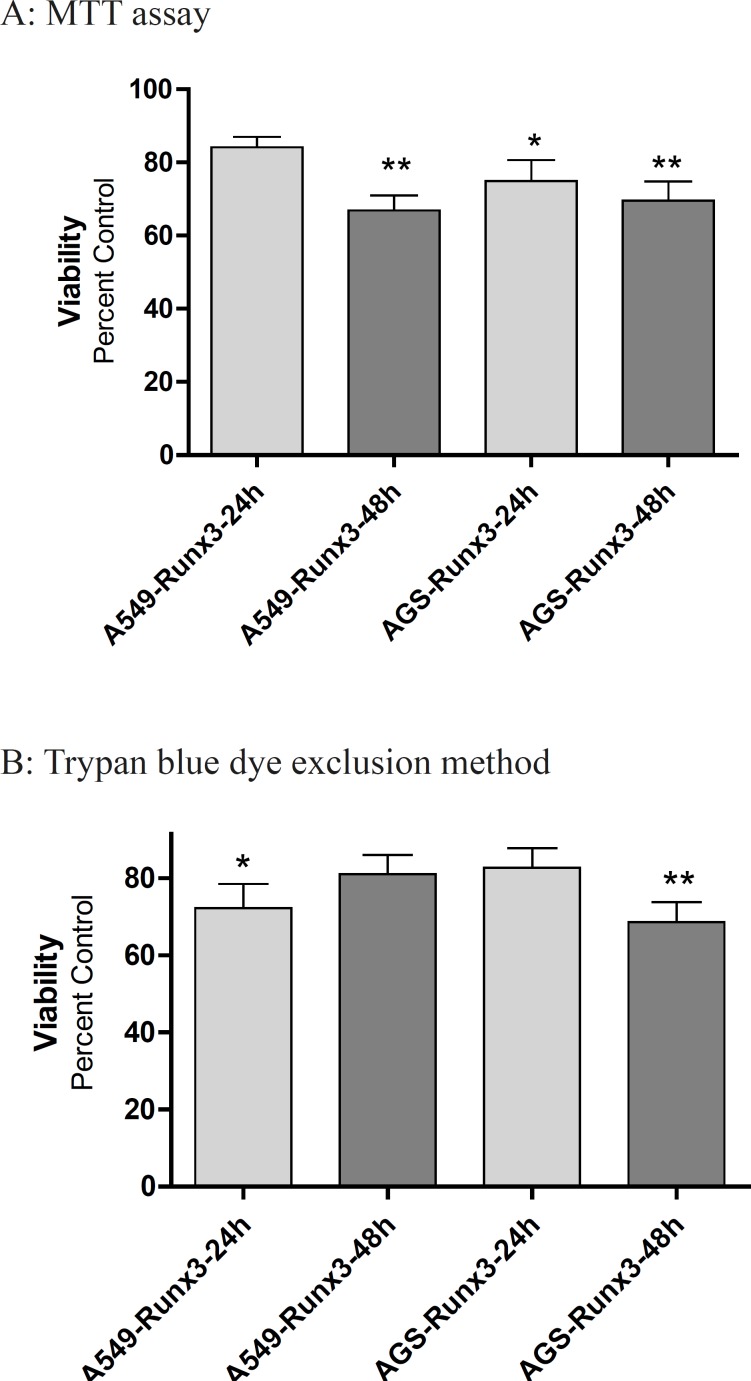
The effect of Runx3 transfection on A549 and AGS cells viability Cells were transfected with Runx3 or empty vector (Control). After 24 and 48 h, the cell viability and proliferation were evaluated by (A) MTT assay (B) and trypan blue. The results are presented as mean ± SE of three independent experiments (n = 3, *p < 0.05, ** p < 0.01

The transfection of GFP-Runx3 inhibited proliferation of AGS and A549 cells within 48 h (Figure 2). According to the MTT results, AGS cells over expressing Runx3 showed 30-40% lower viability within 48 h compared to the vector transfected (Control) cells (p < 0.01; Figure 2A). These results were confirmed by Trypan blue dye exclusion method where 31.4% decrease in live cells were observed in 48 h after the transfection (p < 0.01). In A549 cells, a significant decrease in viability (33.2%, p < 0.01) was observed in 48 h based on the MTT results (Figure 2A).

*RT* *-* *PC Ranalysis of Baxm RNA expression*

**Figure 3:**
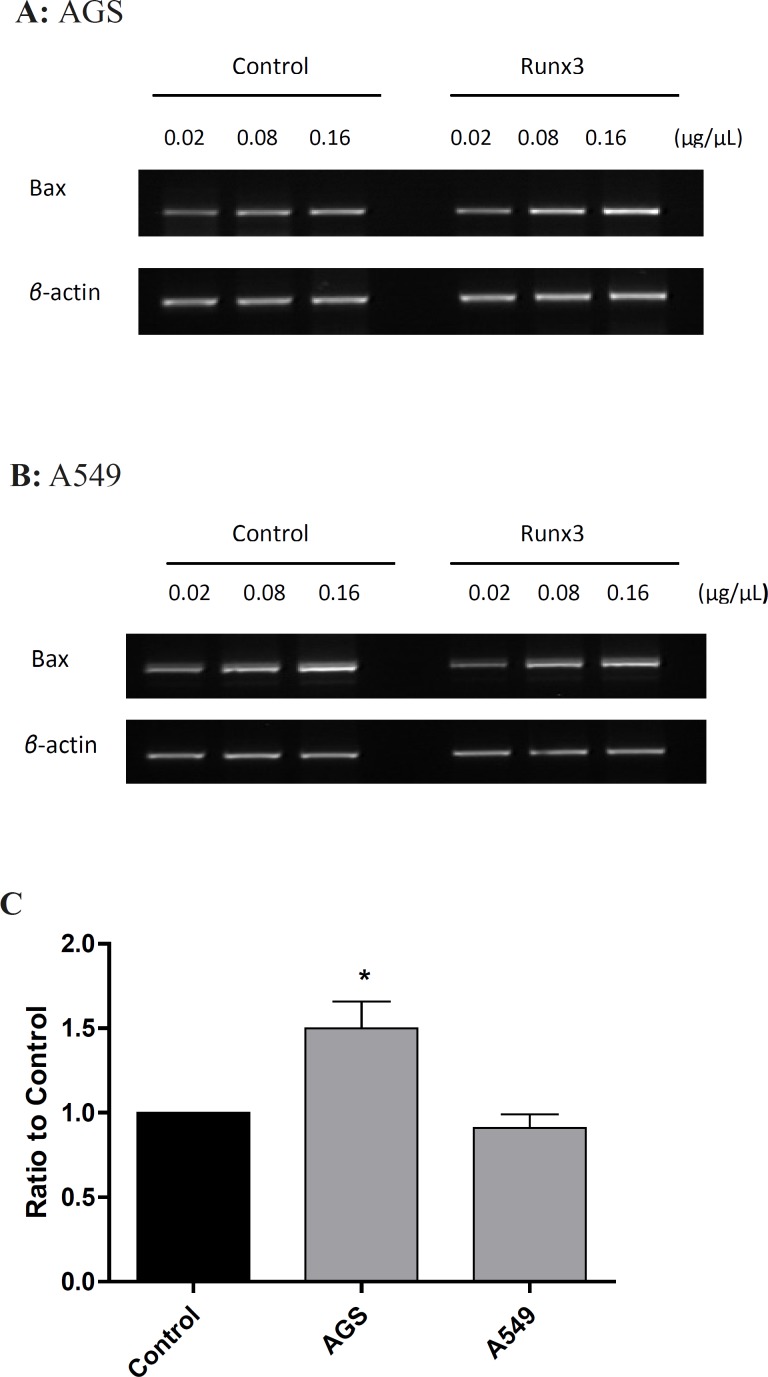
Expression of Bax gene in AGS and A549 cells Cells were transfected with Runx3 or empty vector (Control). RT-PCR was performed on 0.02, 0.08 and 0.16 μg/μL of cDNA with specific primers for Bax in (A) AGS and (B) A549 cells. (C) The gene expression was calculated as ratio to control and presented as mean ± SE of three experiments repeated twice (n = 2, *p < 0.05

To investigate the effect of Runx 3 transfection on Baxm RNA expression, we evaluated the expression of these genes by RT-PC Ranalysis. As shownin Figure 3, Runx3 induced Bax expression in AGS cells (p< 0.05 ) ,suggesting induction of cell death in these cells.

## Discussion

 Runx family of proteins have essential functions in both cell proliferation and differentiation (1, 6) and play roles as proto-oncogenes and tumor suppressors (1). Among them, Runx3 is a tumor suppressor that has antiproliferative effect and induces TGF-*β*-dependent apoptosis (21).

In this study, we have tested the effect of Runx3 expression on proliferation of AGS cells with undetectable protein level of Runx3 and A549 cells with low expression of the protein. We presently show that Runx3 inhibits cell proliferation and viability in both AGS and A549 cells. These findings are in agreement with previous reports indicating that expression of Runx3 will restore TGF-*β* induced apoptosisin biliary track cancer cell, esophageal adenocarcinoma and AGS cells(21-23). It should be mentioned that in both A549 and AGS cells, Runx3, overexpressing by itself, induces cell death. Moreover, our results indicate that Runx3 induces Bax expression in AGS cells (Figure 3). 

It is known that Bax can promote mitochondria-mediated apoptosis in AGS cells (24). Therefore, in AGS cells, Runx3-induced cell death can be mediated via Bax expression. On the other hand, Runx3 expression did not change Bax mRNA level in A549 cells (Figure 3). So far, there have been no reports on the effect of Runx3 expression on Bax induction. It has been reported that the over expression of Runx3 in gastric cancer cells down regulates Bcl-2 and directly activates the promoter activity of Bim to enhance TGF-*β*-dependent apoptosis, therefore, sensitizes cancer cells to chemotherapeutic drugs (18, 25). In addition, it has been reported that Runx2 induces Bax expression in osteosarcoma cells by binding to regulatory domain on the human Bax promoter (26). Hence, it is possible that Runx3 utilizes the same mechanism as Runx2 to induce Bax expression. However, this mechanism is cell dependent since in A549 cells, Runx3 expression could not alter Bax level and perhaps a more complex mechanism is involved in Runx3-induced Bax expression. 

Taken together, Runx3 transfection inhibits cell proliferation in AGS and A549 cells. Moreover, Runx3 expression increases Bax mRNA levels in AGS cells indicating the induction of cell death and cell cycle arrest. However, Runx3 expression fails to induce Bax in A549 cells.
